# The Ongoing Impact of COVID-19 on Pediatric Obesity

**DOI:** 10.3390/pediatric16010013

**Published:** 2024-02-02

**Authors:** Domenico Iacopetta, Alessia Catalano, Jessica Ceramella, Michele Pellegrino, Maria Marra, Elisabetta Scali, Maria Stefania Sinicropi, Stefano Aquaro

**Affiliations:** 1 Department of Pharmacy, Health and Nutritional Sciences, University of Calabria, 87036 Arcavacata di Rende, Italy; domenico.iacopetta@unical.it (D.I.); jessica.ceramella@unical.it (J.C.); michele.pellegrino@unical.it (M.P.); mariamarra1997@gmail.com (M.M.); stefano.aquaro@unical.it (S.A.); 2 Department of Pharmacy-Drug Sciences, University of Bari “Aldo Moro”, 70126 Bari, Italy; 3 Department of Health Sciences, Magna Graecia University, 88100 Catanzaro, Italy; elisabettascali@libero.it

**Keywords:** childhood obesity, pediatric obesity, overweight, COVID-19, pandemic, children

## Abstract

In the developed world, pediatric obesity (PO) has been a major health concern since the last century, and this condition may lead to detrimental life-long physical and mental comorbidities. Currently, its prevalence has increased in low- and middle-income countries and in many high-income countries. Thus, the provision of effective and tailored care for children and their families has become vital. The social consequences of the COVID-19 pandemic are known everywhere, and among these, it has been argued that the COVID-19 pandemic has had a major impact on PO. Overall, the growth of PO over the last decade has been enhanced by the pandemic. During the COVID-19 pandemic, children, adolescents and young adults gained weight as the pediatric population dealt with sedentary lifestyles and changes in food habits. In this review, we want to highlight the impact that the COVID-19 pandemic had on PO.

## 1. Introduction

**Figure 1:**
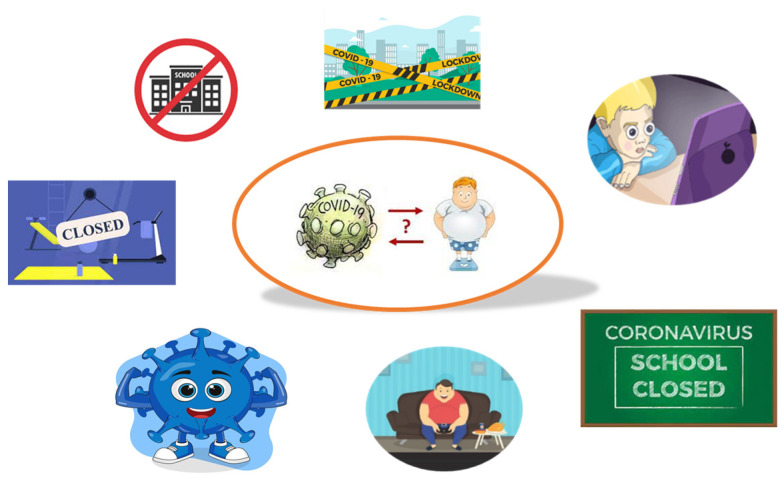
Impact of the COVID-19 pandemic on pediatric obesity.

PO (or childhood obesity) is the most frequent nutritional disorder among children and adolescents around the globe [1]. It affects about 20% of U.S. youth [2]. Over 90% of obesity cases are idiopathic, and less than 10% are associated with genetic and hormonal causes [3]. Several factors, including eating habits, lifestyle, genetics, metabolism, and environment, play a role in the development of obesity. PO is related to a higher risk of several diseases, including diabetes, cardiovascular disorders, stroke, asthma, certain types of tumors later in life, social problems, and depression among youth. In addition, children who are overweight and obese are likely to remain obese into adult age, thus leading to several diseases, including cardiovascular diseases and diabetes, at a younger age. Dysbiosis, that is, the disruption of the natural balance between the gut microbiota and the host, may interfere with metabolic pathways, resulting in the occurrence of obesity and other metabolic disorders [4]. The number of children who are overweight and have PO has increased in recent decades: in 2016, approximately 124 million children and adolescents aged 5–19 were affected by obesity worldwide, and 213 million were affected by overweight [5]. It was estimated that by 2050, about 60% of adult men, about 50% of adult women, and about 25% of children will be affected by obesity. The World Health Organization (WHO) considers PO to be one of the most important health challenges worldwide in the 21st century, with a serious burden on society [6]. PO is studied worldwide across Latin America and the United States [7], Europe [8], Asia [9], Africa [10], and Australia [11], and its prevalence varies amongst diverse countries. In the United States, 17% of children and adolescents were affected by this condition in 2017 [12]. From 2017 to 2020, the prevalence was about 19.7%, affecting approximately 14.7 million children and adolescents [13]. Specifically, the prevalence of obesity was 12.7% among 2–5 years old, 20.7% among 6–11 years old, and 22.2% among 12–19 years old. PO in the U.S. is also more common in certain communities. The prevalence of PO was 26.2% amongst Hispanic children, 24.8% amongst non-Hispanic Black children, 16.6% amongst non-Hispanic White children, and 9.0% amongst non-Hispanic Asian children [14]. Spinelli et al. (2019) [15] reported on a study conducted by the World Health Organization European Childhood Obesity Surveillance Initiative from 36 countries in Northern, Eastern, and Southern Europe and Central Asia. Overall, overweight and/or obesity affected about 28.7% of boys and 26.5% of girls. The incidence of obesity was 1.8% for boys and 1.1% for girls in Tajikistan, whereas it was 21.5% and 19.2%, respectively, in Cyprus. Above 60% of children who are overweight before puberty are also expected to be overweight in early adulthood [16]. Obesity prevention methods, such as healthy lifestyle education for both parents and children, were shown to be important elements in the occurrence of obesity. Multi-component prevention methods that entail the community, school, and family are requested [17]. Unfortunately, the efficacy of PO treatment was mitigated or abolished during the coronavirus disease 2019 (COVID-19) pandemic [18,19,20]. Some authors recently reported the “emerging pediatric obesity epidemic with the COVID-19 pandemic” [21]. Before the pandemic, the incidence of PO had leveled in many high-income countries, even though levels of severe obesity had increased. During the pandemic, weight gain in children was enhanced in several countries. A recent systematic review and meta-analysis by Anderson et al. (2023) [22] evidenced that, during the first year of the pandemic, a marked increase in weight gain and a higher prevalence of obesity in children and adults occurred, even though it was more evident in children. The increased consumption of ultra-processed foods (UPFs), especially sweets, snacks, and baked goods, has been observed [23]. Additionally, for children who are overweight and obese, the impact of lockdown was stronger than that observed for other children; in fact, increasingly sedentary lifestyles, incorrect diets, and social distancing halted the possibility of losing weight (Figure 1). Clear evidence of the aggravation of PO and diabetes mellitus in children during the pandemic was also observed [24]. Obesity and COVID-19 are pandemics that negatively impact the health and well-being of children [24]. A complex and bidirectional correlation between the obesity and COVID-19 pandemics may exist. The clashing of PO and COVID-19 and the consequent modifications to the bioecological environment led children and adolescents to an increased risk of developing obesity and exacerbated the severity of PO [25]. On the other hand, children with PO may undergo a more severe COVID-19 trajectory, sometimes leading to the need for respiratory support [26]. Weihrauch-Blüher recently assessed the evidence that two years after the onset of the pandemic, there is evidence that its adverse effects are not spontaneously reversible [27]. In this review, we analyzed the influence that COVID-19 had on PO and related disorders and summarized the opportunities to curb this disease. This is a narrative review that used Scopus, PubMed, MEDLINE, and ScienceDirect to review the literature on the clinical aspects of PO. A manual search on Google Scholar was utilized to find pertinent papers based on the eligibility criteria in this study. We used Google Scholar to identify cross-sectional surveys and preclinical and clinical studies reporting the prevalence of PO in children and adolescents (≥90% of participants aged ≤18 years). The search criteria considered the occurrence of the association of the following keywords: “pediatric obesity”, “childhood obesity”, “COVID-19”, “Coronavirus Disease-19”, “obese adolescents”, and “overweight”, either in the title and abstract or in the text. Medical journals with low impact factor and/or h index were excluded from this review.

## 2. COVID-19

The COVID-19 pandemic was caused by the pathogen severe acute respiratory syndrome coronavirus 2 (SARS-CoV-2). The pandemic presumably began in December 2019 in Wuhan, China, and then affected many countries around the world, turning into a serious worldwide threat. Up to 13 September 2023, 770,563,467 cases of COVID-19 were diagnosed, and about 7,000,000 deaths were recorded [28]. During these four “long” years, several variants of SARS-CoV-2 were recognized [29]. Vaccines and drugs (antivirals, antimalarials, antibiotics, immunomodulators, angiotensin II receptor blockers, bradykinin B2 receptor antagonists, corticosteroids, and so on) have been used to mitigate the pandemic [30,31]. However, the current emergence of novel viral variants remains a challenge, with reduced protection against infection, as seen with the new Omicron EG.5 variant that exhibits extensive escape from neutralizing antibodies [32,33]. Nevertheless, COVID-19 led to serious outcomes, especially in children and young people [34]. The social outcomes of the COVID-19 pandemic are commonly known [35]. It has rekindled antibacterial resistance, a renovated public health plague in recent times, in adults and children [36], and negatively affected the treatment of several diseases, including cancer, both in terms of screening, diagnosis [37], and treatment [38,39]. The harmful consequences of the disease on mental health and its neurological implications are well known [40,41]. The number of young people in psychiatric emergency care during the chronic period of the pandemic who previously had not required psychiatric contact increased. There was also an increase in the rates of suicidal ideation, thus indicating that youth encountered increased distress during these periods [42]. Moreover, existing data suggest a possible correlation between COVID-19 and early puberty. Obesity, physical activity, mental health, and birth weight are significant risk factors contributing to the early onset of puberty [43]. The worst effects of the COVID-19 pandemic have been evidenced in vulnerable populations [44]. Finally, many children who have recovered from the acute COVID-19 infection can develop post-COVID (also referred to as long COVID) [45,46].

## 3. Pediatric Obesity and Related Diseases

**Figure 2:**
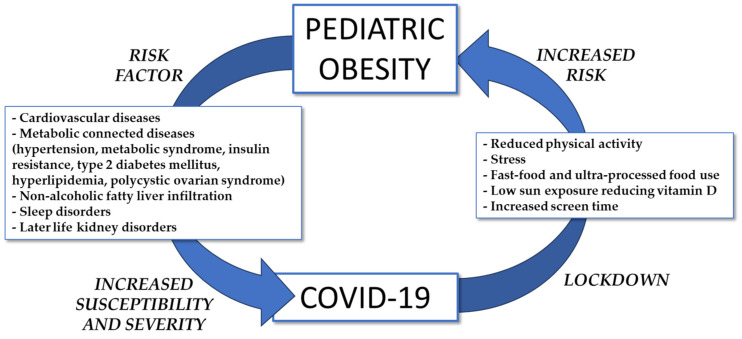
Inter-relationships between pediatric obesity and COVID-19.

The pathogenesis of PO is multifactorial and derives from the relationship between genetic, epigenetic, and environmental determinants [47]. Actually, over 1100 independent genetic loci have been associated with obesity, and there has been significant attention paid to decoding their biological functions and gene–environment interactions. The genetic basis of PO was recently extensively reviewed by Vourdoumpa et al. [48]. Moreover, the autonomic nervous system may also contribute to the distribution of body fat and the development of obesity and its complications. Environmental factors, including built environmental factors (i.e., street connectivity, residential density, access to green space, public transport, bike lanes, sidewalks, and so on) and food environmental factors (i.e., access to convenience stores, supermarkets, fast-food restaurants, fruit and vegetable markets, and so on) may also influence the prevalence of this disease [49]. The inter-relationships between PO and COVID-19 are schematized in Figure 2.

Obesity in children is a significant risk factor for cardiovascular and metabolic connected diseases [50], including hypertension, metabolic syndrome, insulin resistance, type 2 diabetes mellitus, hyperlipidemia, polycystic ovarian syndrome [51], non-alcoholic fatty liver infiltration [52], later-life kidney disorders [53], precocious puberty, and early accelerated growth [54]. There is a close relationship between obesity, hypertension, and COVID-19, which should be taken into consideration due to the long-term cardiovascular risk in children. A cross-sectional study from Song et al. (2023) [55] showed a higher incidence of hypertension in Korean children who were overweight and obese, more prevalent in boys than in girls, during the COVID-19 pandemic. A study conducted by Ortiz-Pinto et al. (2022) [56] evidenced that PO increases the vulnerability to infectious diseases, particularly SARS-CoV-2 infection. The longitudinal childhood obesity study (ELOIN) along with epidemiological surveillance system data from the Community of Madrid (Spain) were used to identify the SARS-CoV-2 infected patients, and then the parameters of the eligible participants, such as their weight, height, and waist circumference, were recorded. General obesity and abdominal obesity were determined according to the WHO-2007 and International Diabetes Federation criteria, respectively, whereas the relative risks for infection were determined using a Poisson regression model. Their results indicated an incidence of SARS-CoV-2 infection equal to 8.6% and the relative risk of SARS-CoV-2 infection of 2.53–2.56 for children 4–9 years old with stable general obesity and/or abdominal obesity. In addition, obesity is a factor in increasing the risk of asthma, one of the most common chronic respiratory diseases, and the importance of diet in asthma development is well recognized. Specific food composition, particularly fat, sugar, and low-quality nutrients, may promote the chronic inflammatory state in asthmatic patients with obesity. Recently, an unbalanced diet or supplementation were identified as ways to ameliorate respiratory symptoms, signs, and therapeutic response ins asthmatic patients in relation to PO. However, weight loss or reduction in asthmatic children with excess weight is an essential target [57]. It is known that COVID-19 infection impacts the small airways in asthmatic children, but it is still debated whether chronic asthma in childhood may be a risk factor for severe COVID-19 infection.

## 4. Pediatric Obesity in the COVID-19 Era

School closures and other restrictions due to the COVID-19 pandemic upset the everyday routine of children and adolescents, resulting in changes in eating behaviors and physical activity. Several papers were reported during the pandemic, suggesting particular attention to these patients [58]. Unhealthy choices of everyday meals likely predominated during the pandemic [59]. The COVID-19 pandemic magnified the vulnerability to both food insecurity and PO in low-income children and families [60]. Additionally, the individuals who were overweight or obese before the pandemic experienced a higher increase in body mass index (BMI) and since that during the COVID-19 pandemic traditional medical visits were mandatorily replaced by virtual ones, many children involved in weight management programs experienced interruptions to their medical care, which probably negatively impacted weight management [61]. Other factors that negatively influenced the lives and weight management of children and adolescents during the pandemic, mostly those living in disadvantaged families, include difficulties due to the loss of their parents’ income, new expenses for their care (internet and devices, for example), lack of in-person education, a decline in mental well-being, and a higher risk of domestic maltreatment [62]. The impact of the COVID-19 pandemic on family well-being has been recently reviewed [63]. A study carried out in Finland [64] found that earned income losses during the pandemic affected all families with children, with the most detrimental economic impact in May 2020. Further devastating effects of the pandemic on adolescents were embodied in the loss of a parent or caregiver, decreased access to resources, including school support and health resources, and the loss of a parent’s employment [65]. These losses have influenced the social, emotional, and economic well-being of children and their families [66]. Moreover, the COVID-19 pandemic increased the frequency of child maltreatment [67]. The consequences of stress related to social isolation and anxiety generated by the pandemic on mental and physical health are also collateral effects [68]. It is noteworthy that students are likely most anxious about online learning, mainly due to challenges in internet usage, as reported by Nutsugbodo et al. (2023) [69] in a study carried out in Ghana. It is noteworthy that disparities among different demographic groups during the COVID-19 pandemic were observed [70]. From 2018 to 2020, there was a significant increase in BMI, with an overall increase in patients with overweight and obesity in 2020. In a study carried out in Israel during the first six months of the pandemic, there was a considerable increase in obesity in preschoolers [71]. Shalitin et al. (2022) [72] conducted a large retrospective cohort study based on data obtained from Clalit Health Services in Israel, which included persons aged from 2 to 20 years old and their sociodemographic, anthropometric, clinical parameters and BMI measurements in the pre-pandemic and pandemic periods. The results indicated a substantial weight gain correlated with the COVID-19 pandemic, mostly in children aged from 2 to 6 years old, and that the BMI increased significantly in underweight children and decreased in those who were overweight and obese. The negative impact of the pandemic on the BMI of children with obesity was also evidenced in a retrospective study [73] of a Swiss population, and it was higher than in normal-weight children. An observational study by Patel et al. (2022) [61] carried out on patients from the SickKids Team Obesity Management Program (STOMP) from November 2018 to July 2021 in Ontario, Canada, suggested that the COVID-19 pandemic likely lowered the health impacts of a program for weight management, specifically in males, resulting in higher body weight and BMI. A recent study by Hu et al. (2022) [74] on weight-related health behaviors, such as physical activity, screen time, sleep, diet, and “social determinants of health” (SDoH), including food insecurity, income/childcare, and caregivers’ perceived stress, was published referring to a survey administered from August to October 2020 to caregivers of 2–17 years old and adolescents aged between 13 and 17 with a BMI ≥85th percentile. The surveys were completed by 129 caregivers and 34 adolescents. In comparison with the pre-pandemic period, caregivers reported a decrease in youth moderate/vigorous physical activity (−87.4 min/week) and increased recreational screen time (2.5 h/day). Fewer had regular bedtimes (89% before and 44% during the pandemic). and more ate most meals with television (16% and 36%, before and during the pandemic, respectively). Food insecurity increased from 27% to 43%; 45% stated lower household income, and caregivers with moderate/high perceived stress scale scores increased from 43 to 64%. It is worthy of note that not all children experienced the same increase in obesity during the COVID-19 pandemic. Mayne et al. (2023) [75] reported that children who lived in greener neighborhoods underwent smaller rises in obesity during the pandemic than those who lived in less green neighborhoods, even though differences in terms of urbanicity were also observed. A large-scale study assessed the obesity incidence and BMI of children and adolescents from diverse regions of China. From 2017 to 2021, the incidence of obesity and overweight was recorded at a stable level but, again, the 2020 lockdown dramatically impacted obesity and weight gain, mostly in the northern region of China and after the age of four [76]. Another prospective cross-sectional and school-based study [77] analyzed the standardized BMI (zBMI) and prevalence of obesity data from 2014 to 2020 among children and adolescents 6–17 years old in Changshu City (China), comparing age-specific subgroups and sexes. The results indicated that the overall zBMI mean and the incidence of obesity drastically increased from 0.29 in 2019 to 0.45 in 2020 and from 10.38% in 2017 to 12.77% in 2020, respectively. A fundamental role in the weight-related behaviors of children with obesity during the pandemic was played by parents. Parents did not perceive their child’s weight exactly; furthermore, low levels of parental concern for their child’s weight were found [78]. An interesting study was carried out by Neshteruk et al. (Clinical Trial: NCT03339440) [79] on Black (46%) and Hispanic (39%) children, with a mean age of 9.7 (±2.8) years, from low-income families (62%). In this study, most parents of the participants were represented by mothers (88%). There were discrepancies in the perception of physical activity levels: some parents evidenced a rise in activity or maintenance of activity levels to enhance outdoor time, whereas other parents reported a reduction due to the lack of outdoor time, school, and structured activities. Key dietary changes included increased snacking and more meals prepared and consumed at home. There was a change in sleep schedules: children went to bed and woke up later, and there was a rise in leisure-based screen time. A multicenter retrospective study was carried out on 7150 children aged 3–19 years in New York City to evaluate if the negative impact of the pandemic on weight in children persisted after public health restrictions were reduced. The authors demonstrated that excessive weight gain in 2020 was not reversed by 2021 [80]. An online cross-sectional study [81] was conducted among the parents of Italian children (between 5 and 9 years old) and adolescents (between 10 and 14 years old) based on a self-administered questionnaire that included weight and height measurements and dietary habit changes during the lockdown (from March to June 2020). Different changes in eating habits were recorded, particularly sweet, packaged snack consumption, which increased by about 34%; processed meat, which increased by about 25%; and bread, pizza, and bakery products, which increased by about 47%. However, an increase in vegetable, fresh fruit, and legume intake of 19% was also registered, together with a decrease in sweet beverage and candy intake. About 60% of the people involved in the study gained body weight, mostly adolescents with respect to children (67% vs. 55%); in the latter case, this was associated with increased body height and dairy products and sweet, packaged snack consumption. In adolescents, the increased weight was instead associated with a higher consumption of comfort foods and processed meat. A recent study by Salman et al. (2023) [82] compared the incidence of overweight and/or obesity in school-aged children in Isparta, Turkiye, during the pandemic and compared the results with those obtained in previous studies (2005, 2009, and 2014). The incidence of overweight and obesity increased during the pandemic. Moreover, it was noted that, during the pandemic, PO occurred most often before the age of 11 years, while in previous studies, it occurred before 5 years of age. In a recent study carried out in Sweden on preschool children, a rise in overweight and obesity was noted among 3 and 4 year-old-children, and this finding was exacerbated in children attending child health centers in areas with lower socioeconomic status [83]. Finally, PO is related to long COVID. Obese adolescents are at a greater risk for long COVID [84]. 

## 5. Management of Pediatric Obesity and Strategies for Pediatric Obesity Treatment during the COVID-19 Pandemic

The COVID-19 pandemic led to economic trouble, school closures, lockdowns, limited physical activity, and enhanced food insecurity for a lot of families [85]. The American Academy of Pediatrics published a guide for pediatricians in December 2020 to help children and families in the management of healthy lifestyles and obesity during the pandemic [86]. It contained recommendations for virtual activities promoting enhanced physical activity, the connection between families and nutritious meals proposed through community settings, and ways to remain safe and active outside of the home, such as going to parks, biking, and walking [87]. The first attempt to prevent COVID-19 was achieved through education for hand hygiene using alcohol-based [88] or antimicrobial-based hand sanitizers containing endocrine-disrupting chemicals (EDCs), such as bisphenol A, phthalates, and polychlorinated biphenyls (triclosan and triclocarban) [89,90]. The use of EDCs was demonstrated to induce PO [91]. Exposure to EDCs has been causally related to obesity in model organisms and obesity occurrence in humans. Indeed, EDCs are defined as “obesogens” as they are able to promote adipogenesis and obesity via several mechanisms [92,93]. At the beginning of the COVID-19 pandemic, the need for telehealth and e-health was pointed out [94]. During this period, telemedicine, such as telephone calls, video calls, and data platforms, was implemented in order to ensure follow-up visits and medical consultations by healthcare providers. The pandemic caused the need to promote telemedicine at lightning speed in several clinical practices; thus, there was insufficient time to evaluate clinician acceptance, child and caregiver acceptance, and training [95]. At first, the management of obesity through telemedicine encountered patient- and healthcare-specific barriers [96]. Enhanced awareness of these barriers then allowed physicians to well understand patient interactions via virtual medical visits [97]. Good results were generally obtained for obese and diabetic children regarding metabolic control and weight management in centers that performed broad telemedicine services, even though inequalities resulted from the spread of technological infrastructure and mobile or internet availability for several patients [98]. A systematic review of randomized controlled trials by Shah and Badawy (2021) [99] evidenced that telemedicine services for the general public and pediatric care were similar or better than those provided in person. A narrative review recently described the role of telehealth and tele-exercise as useful tools in the management of PO during the pandemic. The enhanced use of technology led clinicians, teachers, and trainers to support relationships with children with obesity to reduce sedentary behaviors and the associated health risks [100]. A study on 156 patients regarding the telehealth intervention was carried out in an outpatient clinic in South Italy. Telehealth determined the maintenance of baseline weight status in participants, with an increase in fat-free mass [101]. A recent study highlighted that telehealth use changes on the basis of age and ethnicity among patients with obesity [102]. Chaves et al. (2022) [103] have recently reported a commentary on the impact of the pandemic on PO, specifically on the return to a healthy new “normal”. This paper is essentially for parents and families from a psychological point of view, encouraging children to increase their gratitude and praise toward themselves and one another. Families are encouraged to celebrate victories, focus on healthy behaviors, and not persevere through what may not be right. Psychological factors, including distorted body image and negative mood, play a main role in eating behaviors and obesity; thus, stand-alone psychological interventions or interventions adjuvanted with traditional behavioral strategies represented an important target. Moreover, some other literature studies are reported in the literature concerning psychological factors and interventions as well as healthy behaviors [104,105,106]. One of the most used approaches is Cognitive Behavioral Therapy (CBT), which focuses on the modification of behaviors and cognitive techniques in order to modify dysfunctional cognitions, resulting in improved food habits and quality of life [107]. In addition, an emerging area of research, namely Acceptance and Commitment Therapy (ACT), was shown to be particularly effective in the management of adolescent obesity and is based on the acceptance of internal experiences, such as food cravings, rather than rejection [108]. Finally, the management of obesity-related hypertension requires a multifaceted approach that targets lifestyle modifications and weight loss. The timely identification of metabolic complications is essential and requires pharmacologic treatment to prevent cardiovascular diseases [109].

Different strategies were proposed to improve the management of PO. However, the reluctance of parents to be involved in PO prevention activities, a lack of adequate knowledge, and financial concerns are the most commonly described obstacles in this field [110]. Recently, the major options for PO management, such as lifestyle modification approaches and pharmacological therapies, as well as the treatment indications for the general practitioner, have been reviewed [111]. Medications, including orlistat, phentermine, phentermine/topiramate, and GLP1 receptor agonists (liraglutide and semaglutide), have also been approved for patients who are 12 years old and older. Bariatric Roux-en-Y gastric bypass and vertical sleeve gastrectomy for adolescents with severe obesity have also been used [112]. Motivational interviewing techniques are often recommended and used by clinicians to promote healthy behavior [113]. mHealth technology utilizes mobile phones with several mobile applications (apps) available for iPhone/iPad for pediatric weight loss, healthy eating, and physical activity [114]. However, the use of these apps is controversial, and most apps do not target parents and/or families and focus only on the individual child. The multi-component approach described by Griffiths et al. (2021) [115] for the treatment of PO, including behavioral approaches to alter diet and sedentary behavior, failed in children, while it showed good results in adults. Indeed, children did not follow dietary recommendations. The management of screen time, which is a component of sedentary behaviors to be avoided, is also particularly effective in young children. Interventions that immediately decrease screen time include better parental control, the e-monitoring of time on digital devices, and the removal of electronic games from the bedroom [116]. However, screen time increased during the pandemic. The ENDORSE project makes use of mhealth technologies, artificial intelligence, and serious games for connecting healthcare professionals, children, and their parents with the objective of delivering coordinated services to curb PO. It comprises activity trackers, a serious mobile game for children, and mobile apps for parents and healthcare professionals. Clinical trials are ongoing to explore the effectiveness and sustainability of the ENDORSE platform [117]. Cuda et al. (2022) [118] described new and promising medical and surgical therapies and screening tests for rare genetic causes of obesity, focusing on the impact of COVID-19 in pediatric patients. The most effective and durable treatment for obesity and related complications is metabolic and bariatric surgery, which can improve the quality of life from a psychophysical point of view. However, this practice is reserved for the most severe forms of obesity and in the presence of heavy complications [119]. The global, multicentre, and observational cohort study conducted by Singhal et al. (2021) [120] between May and October 2020 in 24 countries indicated that metabolic and bariatric surgery may be a safe procedure when precautionary procedures, such as pre-operative testing, are used; indeed, the 30-day morbidity rates were pretty unchanged with respect to those reported before the pandemic. Recent studies have addressed endoscopic sleeve gastroplasty (ESG). Although it is currently not FDA-approved for children, it has yielded interesting results. Alqahtani et al. (2019) reported the first study regarding the use of ESG on 109 pediatric patients with PO [121]. The authors observed that the procedure was safe and effective in children and adolescents with obesity, producing significant and long-term weight loss. It is noteworthy that weight loss goes along with a rise in telomere length, and an initial longer telomere length may be a predictor for superior response to treatment. Changes in telomere length may also be associated with complications related to obesity, including metabolic syndrome and diabetes mellitus. Thus, telomere length at the onset of an intervention may predict modifications in weight reduction, improve fasting glucose levels, and decrease inflammatory biomarkers after treatment [122]. 

## 6. Changes in the Prevalence of Pediatric Obesity after COVID-19 Pandemic

Most studies report that the COVID-19 pandemic worsened PO. Anderson et al. (2023) [22] evidenced that, during the first year of the pandemic, there was a higher prevalence of PO. Littlejohn et al. (2023) [13] write that PO was made worse by the pandemic, with the pandemic serving as an accelerator worldwide. Piątkowska-Chmiel et al. (2023) [123] reported a rise in the incidence of overweight in children and PO during the COVID-19 pandemic due to chronic stress. Choi et al. (2023) [124] recently reported a cross-sectional study using the Korean National Health and Nutrition Examination Surveys (KNHANES), concluding that the prevalence of PO and metabolic syndrome among children and adolescents has risen after the COVID-19 outbreak. Hawkins [125] investigated the effects of the COVID-19 pandemic on pediatric body mass index and health status. Multiple studies have already demonstrated a relationship between increased obesity and the pandemic. Chang et al. (2021) [126] reported a systematic review and meta-analysis following the Preferred Reporting Items for Systematic Reviews and Meta-analysis (PRISMA) statement based on four online databases (EMBASE, Medline, the Cochrane Library, and CINAHL), concluding that the prevalence of obesity and overweight increased after the COVID-19 pandemic. The study by Vogel et al. (2022) [127] compared the trends in the 3-month change in BMI in the period 2005–2019 and the respective changes from 2019 to 2020 (pre-pandemic and after the onset of anti-pandemic measures, respectively) in more than 150,000 children (9689 during the pandemic period), which corresponds approximately to the first lockdown phase in Germany. The results indicated a positive trend in weight gain patterns, especially within the group of children with PO. Koebnick et al. (2023) [128] reported that the COVID-19 pandemic lockdown led to excess body weight, particularly for Black and Hispanic youth, which varied depending on social factors related to race and ethnicity. Long et al. (2023) [129] examined the impact of the COVID-19 kindergarten closure from 2018 to 2021 on overweight and obesity among 3–7 year-old children. It was found that the rates of overweight and obesity significantly increased in 2020 after a 5-month kindergarten closure. The study carried out by Pinheiro et al. (2023) [130] in a Portuguese hospital on 422 children and adolescents (aged between 10 and 15 years) assesses that the COVID-19 pandemic caused weight gain in children, enhancing the prevalence of overweight and PO. However, the first lockdown period alone accounted for the ascertained results, and the following period was characterized by a gradual reduction in body mass index, though not sufficient to reach pre-pandemic levels. Woolford et al. [131] reported a retrospective cohort study of more than 190,000 U.S. children who had at least one documented BMI measurement before and one during the first year of the pandemic. Obesity rates increased compared to pre-pandemic levels among all age groups (specifically, the rise was 19–26% for 5–11 years old, 19–23% for 12–15 years old, and 18–20% for 16–17 years old), also leading to acquired atherosclerotic cardiovascular disease (ASCVD) risk factors in children and adolescents [132]. Lower physical activity, higher screen time, and higher consumption of unhealthy foods were observed, contributing to an increase in preexisting disparities in obesity rates among race and/or ethnic and income groups [133].

Disparities in PO have often been described. Jenssen et al. (2021) [134] reported already alarming disparities in obesity rates among children aged between 2 and 17, which have risen since the onset of the COVID-19 pandemic. Before the pandemic, external epidemiological data showed that obesity rates were higher in non-Hispanic Black and Hispanic youth than in non-Hispanic white or Asian American children and adolescents [135], and obesity rates diminished in well-educated and more affluent households but continued to increase for others [136]. During the pandemic, preexisting disparities in obesity in terms of race and ethnicity, insurance, and neighborhood socioeconomic status were increased. Efforts to reduce COVID-19 transmission have likely contributed to exacerbating PO. Dietz (2023) [137] compared the prevalence of the rise in PO during the COVID-19 lockdown with the annual rise in the National Health and Nutrition Examination Survey (NHANES). The changes in prevalence among elementary school children observed in two population-based surveys were 28–63 times higher than the annual changes observed in NHANES. Increases in Black and Hispanic youth were greater than those in White children and adolescents. The net impact of the COVID-19 lockdown increased the disparities in obesity in this age group.

However, some authors reported an opposite tendency after the COVID-19 pandemic, that is, a reduction in PO following the COVID-19 pandemic. Ban et al. (2023) [138] investigated trends in BMI, overweight, and obesity among 1,111,300 Korean adolescents (mean age: 15.04 years) from 2005 to 2021, including the COVID-19 pandemic. The prevalence of overweight and obesity was 13.1% between 2005 and 2007 and 23.4% in 2021. The mean BMI and prevalence of obesity and overweight have gradually risen over the past 17 years; however, the extent of change in mean BMI and in the prevalence of obesity and overweight during the COVID-19 pandemic (2020–2021) was distinctly less than before (2005–2019). Other authors found differences in the prevalence of PO after the COVID-19 pandemic based on the age of the children analyzed. A study carried out in Japan by Takaya et al. (2023) [139] was aimed at analyzing several data, including PO in Osaka elementary and junior high schools for 4 years (2018–2021) by comparing the pre-pandemic, pandemic lockdown, and post-lockdown periods (2018–2019, 2019–2020 and 2020–2021, respectively). Obesity rates in elementary school students aged 6–12 years were considerably higher during the lockdown than in 2019, especially in boys. In junior high school students aged 12–15 years, the rates of obesity and underweight reduced in 2020. However, these rates rebounded and increased in 2021 when the lockdown was removed. In conclusion, during the COVID-19 pandemic lockdown, elementary school students gained weight, while junior high school students lost weight. The lockdown implemented during the COVID-19 pandemic had an unfavorable effect on weight gain, especially in young school-age children. A cross-sectional survey was performed in Germany in the period April/May 2022 and compared to the one performed in 2020 in children aged 3–17 years [27]. Every sixth child has increased in body weight in an unhealthy manner since the beginning of the COVID-19 pandemic, and this was most frequent in children from families with lower household income and with preexisting overweight or obesity. Negative health effects related to the COVID-19 pandemic were mainly observed in children aged 10–12 years.

## 7. Conclusions

PO is increasing at alarming rates worldwide, and its rates increased enormously during the COVID-19 pandemic. The data collected from different countries evidenced an increase in weight gain among children and adolescents during the pandemic due to COVID-19-related lockdown measures. Only a few authors report the opposite. Several factors, such as lower physical activity, increased screen time, dietary intake changes, food insecurity, and augmented family and individual stress, have contributed to this phenomenon. Serious changes in health behaviors among children with overweight and obesity may enhance the occurrence of obesity-related comorbidities and exacerbate health disparities. The COVID-19 pandemic dramatically highlighted many failings, exacerbating the already existing social and technological disparities, and focused attention on the need to deliver suitable interventions remotely. There is a compelling need to enlarge access to effective interventions for children with overweight and PO that address psychosocial stressors. It is unclear whether the prevalence of PO may decline in the post-pandemic era, and the ongoing impacts of the COVID-19 pandemic may be attenuated. However, according to recent studies, the presence of evidence showing the long-term impacts of COVID-19 persisted despite changes in the prevalence of PO have been recently evidenced. It remains to be seen whether this trend will be confirmed and continue in the future.

## Data Availability

Not applicable.
